# Equation for analyzing the peak power in aquatic environment: An alternative for olympic rowing athletes

**DOI:** 10.1371/journal.pone.0243157

**Published:** 2020-12-17

**Authors:** Paulo Francisco de Almeida-Neto, Luíz Felipe da Silva, Dihogo Gama de Matos, Ian Jeffreys, Tatianny de Macedo Cesário, Rui Barboza Neto, Wagner de Almeida Barbosa, Felipe J. Aidar, Paulo Moreira Silva Dantas, Breno Guilherme de Araújo Tinoco Cabral

**Affiliations:** 1 Department of Physical Education, Federal University of Rio Grande do Norte, Natal, Brazil; 2 Research Center in Sports Sciences, Health Sciences and Human Development, CIDESD, Trás dos Montes and Alto Douro University, Vila Real, Portugal; 3 Group of Studies and Research of Performance, Sport, Healt and Paralympic Sports GEPEPS, The Federal University of Sergipe—UFS, São Cristovão, Sergipe, Brazil; 4 Faculty of Life Sciences and Education, University of South Wales, Pontypridd, Wales, United Kingdom of Great Britain and Northern Ireland; 5 Department of Theoretical and Experimental Physics—Federal University of Rio Grande do Norte, UFRN, Natal, RN, Brazil; 6 Department of Physical Education, Federal University of Sergipe—UFS, São Cristovão, Sergipe, Brazil; 7 Graduate Program in Master’s Level at Department of Physical Education, Federal University of Sergipe—UFS, São Cristovão, Sergipe, Brazi; 8 Program of Physiological Science, Federal University of Sergipe—UFS, São Cristovão, Sergipe, Brazil; Universidade do Vale do Rio dos Sinos, BRAZIL

## Abstract

**Purpose:**

To develop an equation to provide the peak power (PP) through a specific stimulus performed in an aquatic environment, as well as to correlate morphological, anthropometric and strength variables with rowing performance.

**Methods:**

The sample consisted of 16 elite young rowing athletes of both sexes (15.7 ± 1.21 years). The strength of upper limbs and lower limbs was verified. To analyze the PP, a 100-m Sprint test was performed on an indoor rowing type ergometer, and after a 72-hour wash-out, the test was repeated in an aquatic environment on a vessel equipped with a global position system. Body composition was analyzed by examining bone densitometry with an X-ray source and maturation was verified by anthropometry.

**Results:**

The tests for water sprint and indoor rowing showed significant reliability (ICC = 0.695; p = 0.0007). The PP aquatic showed reliability with that acquired in indoor rowing (ICC = 0.897; p<0.0001) and was related to maturation (p<0.05). The morphology, anthropometry and strength of the upper limbs were related to the sprint and peak strength in both tests (p<0.05).

**Conclusion:**

The equation for the PP in aquatic environment presented by the present study is highly reliable with an indoor ergometer digital ergometer.

## Introduction

The Olympic sport of Rowing is contested over a 2000m course and requires a mixture of aerobic and anaerobic power [1]. Times to complete the course are influenced by factors including the crew number, the competition classification (openweight or lightweight), sculling (2 oars) or rowing (1 oar) and the sex of the participants [1]. Rowing is extremely demanding physiologically, involving a high degree of power and endurance, whilst also requiring rowers to be highly technically proficient [2]. Ultimately, the aim is to complete the course in as short a time as possible and this requires the ability to exert high forces for a sustained period [1].

Importantly, rowing is a modality that directly interacts with the aquatic environment, and thus relying on how a rower’s capacities interact with the environment to produce effective propulsion [3, 4]. In this sense, the force exerted by the rower against the water resistance is a constant reality of the sport, and the training prescription must be precise and take into account all the variables that impact upon rowing performance (i.e., physical, technical and environmental characteristics). For this reason, specific tests are recommended to provide the best possible basis for prescribing the training of athletes and rowing teams [5].

Thus, performing simulations of the official competitions of the sport can be of great value for the evaluation and advancement of the athletes' performance [2, 6]. In addition, given the importance of force capacities, anthropometric variables (i.e., weight, height, length of legs and body span) and muscular strength of the trunk and upper and lower limbs are also associated with the performance of rowers [2, 6–8]. To further support this relationship, Durkalec-Michalski et al., [9] stated that muscle power and body mass are determinants for the specific performance of rowers during official tests and competitions.

Anthropometric variables (i.e., height, limb length, etc.) cannot be modulated by sports training [2, 5–9]. However, these variables influence the “biomechanical levers” of the human body during the production of muscle strength [2, 5–9]. This fact can be interesting during the selection process of young sports talents in relation to the specificity of force production in rowing [2, 5–9].

In addition, the analysis of specific performance in an aquatic environment can also be useful during the talent selection process, as well as for the daily training of rowing athletes [10, 11]. A tool used for this purpose is a digital indoor paddle ergometer, as it is considered to be of a high standard for specific assessments in the modality: it is easy to use, it can simulate water resistance, it provides data on the test time in seconds and peak power (i.e., power consumption per second in watts) and some models even manage to simulate the balance of the vessels [12–14]. Among the data provided by the equipment, the peak power is used by rowing coaches as a parameter for monitoring and prescribing training in the modality, as it is understood that the closer an athlete is to their maximum strength peak, the better the quality of the displacement of the vessel in an aquatic environment [15, 16].

In this sense, it is necessary to trace the individual stroke profile of each subject in order to evaluate performance and prescribe appropriate training interventions. It is worth mentioning that the rowers can be classified according to the characteristics of the peak power developed by the force generated by the movement of the paddle blades, or they can classify the stroke patterns in “stroke” (characterized by generating a peak of greater force in the first half of the drive) and bow (characterized by generating a greater peak of force in the second half of the drive) [9, 11]. Therefore, each profile will demand a different emphasis in relation to the training focus (i.e., technical, biomechanical aspects, strength development, etc.) [9, 11].

On the other hand, the probability of the peak power generated in indoor rowing overestimating or underestimating the real effort made in the aquatic environment can lead to a misclassification of the athletes' paddling profile [6, 17]. Therefore, it is not yet clear in the literature what is the best way to estimate the peak power in an aquatic environment [11]. Thus, this research aimed to develop an equation to provide the peak power in rowers through a specific stimulus performed in an aquatic environment, as well as to correlate morphological, anthropometric and strength variables with the rowers' performance. Thus, the present study hypothesized that it may be possible to measure the peak power in an aquatic environment through a mathematical model based on the displacement time of the rowers' vessel.

## Methods

### Subjects

The study was a cross-sectional study with a sample of 16 young rowing athletes (national level competitors from the team of new talents included in the top 20 in the positions between 7th and 12th place (final B) and between 13th and 19th place (final C)) of both sexes with an average age of 15.7 ± 1.21 (81% male and 19% female). Using the studies by Riechman et al., [18] and Cataldo et al., [19], the sample number was established a priori (considering the sprint test performance variable) by adopting an effect size = 0.80 an α = 0.05 and a β = 0.80. For a sample of 16 subjects, the calculated sample power was 0.88.

As inclusion criteria: (i) Be affiliated to an official federation of the sport; (ii) the volunteers should have undertaken a training routine for two years with a minimum training frequency of six times a week (90 min./session); (iii) Do not use any type of substance that could exert any type of ergogenic effect (i.e. food supplements like creatine, caffeine and taurine; use of illegal drugs for performance enhancement as anabolic steroids); (iv) Do not present osteomioarticular lesions (i.e., injuries to bone tissues and or joints), or muscle injuries in the last six months. Athletes who presented some type of limitation to perform the proposed physical tests were excluded from the study.

### Ethics

The research was analyzed and approved by the Research Ethics Committee—of the Federal University of Rio Grande do Norte (CAAE: 15865619.7.0000.5537. Opinion: 3.552.010), which respectfully follows the ethical principles contained in the Declaration of Helsinki. Signed, informed consent (TALE and ICF) were obtained from the participants and their respective legal guardians in accordance with the Resolution 466/12 of the Ministry of Health (Brazil). This study complied with all the international requirements and standards of the STROBE checklist for observational studies [20].

### Procedures

**Fig 1:**
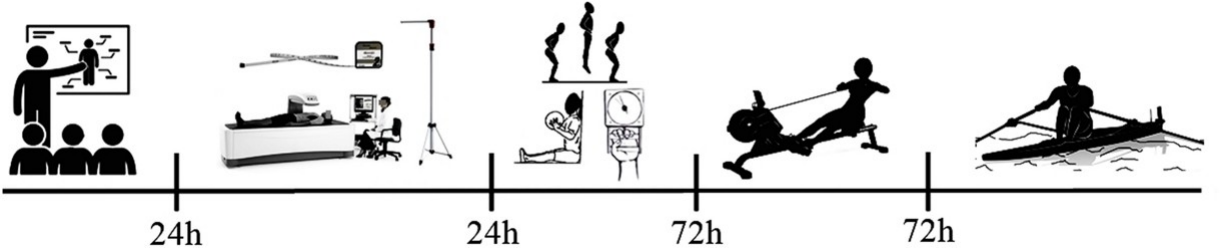
Study design.

Fig 1 reports, through illustrations, the segment that occurred in the study design. In this sense, data collection took place over 5 days according to the following topics: (i) explanations about the research; (ii) anthropometric and body composition tests; (iii) neuromotor tests referring to the muscular strength of the upper and lower limbs (iv) test carried out on an indoor rowing digital ergometer, (v) realization of the specific performance protocol proposed by the present research.

It is worth mentioning that the athletes did not have access to their results during the research. Thus, the protocols were performed by three different evaluators. All evaluators were physical education professionals with experience in the application of the tests and had previous training by the Brazilian Rowing Confederation. In this way, the evaluator “**A**” performed the anthropometry and muscle strength tests, evaluator “**B**” performed the body composition test and evaluator “**C**” applied the specific performance tests.

#### Analysis of anthropometry and body composition

Anthropometric assessments were based on the ISAK protocol and were performed by an experienced anthropometrist (International Society of the Advancement of Kinanthropometry) [21]. Body mass was measured using a digital scale with a variation of 0.10 kg (FILIZOLA^®^, São Paulo, Brazil). Standing and sitting height were assessed by a stadiometer with a precision of 0.01 cm (SANNY^®^, São Paulo, Brazil). The perimeter was measured with an anthropometric tape (SANNY^®^, São Paulo, Brazil). The chronological age in years was calculated from the date of birth of the analyzed subject in relation to the day of the evaluation [5]. Body composition was analyzed by dual energy X-ray emission densitometry (DXA) (LUNAR^®^ / GE PRODIGY—LNR 41,990, Washington, United States), this procedure is considered the gold standard for measuring body composition [16].

#### Somatic maturation assessment

It has recently been shown by our group that the stages of puberty influence the production of muscle strength in young rowing athletes [3]. Given the assumption, maturation could be a significant variable for the analysis of peak power in the present study. Thus, in the present study somatic maturation was calculated using the predictive equations of Mirwald et al., [22]. The formula measures and classifies the peak height velocity (PHV) in relation to chronological age, using anthropometric variables:

**Table:** 

PHV in male sex = – 9.236 + [0.0002708 * (Leg length* Trunk Height)] + [–0.001663 * (Age* Leg length)] + [0.007216 * (Age* Trunk Height)] + [0.02292 * (Weight/ Stature) * 100]
PHV in female sex = – 9.376 + [0.0001882 * (Leg length * Trunk Height)] + [0.0022 * (Age * Leg length)] + [0.005841 * (Age* Trunk Height)] –[0.002658 * (Age* Weight)] + [0,07693 * (Weight/ Stature) * 100]

Once years from PHV is estimated, representing a maturity offset, individuals can be grouped into three maturity statuses: 1) Pre-PHV = were maturity offset <-1; 2) circum-PHV = were maturity offset >-1 and <+1; and 3) Post- PHV = were maturity offset >+1. The method has a strong validation with the pediatric longitudinal monitoring of somatic maturation (male sex: r = 0.959; r^2^ = 0.920; p <0.05; feminine sex: r = 0.953; r^2^ = 0.910; p <0.05) [22].

#### Analysis of neuromuscular performance of the upper limbs

The strength of upper limbs was analyzed using the medicineball test [23]. The participant was seated with his back against a wall and his knees extended. At the evaluator's signal, a medicineball (Ax Sports^®^, Tangará, Brazil) with a mass of 2 kg positioned at the height of the sternum with the elbows flexed, was thrown horizontally from the elbow extension using both hands and the aid of trunk movement was not allowed. It should be noted that the medicineball was wrapped with magnesium chalk powder, and that after the launch the horizontal distance at the first contact of the medicineball with the ground was taken as the result of the test [23]. The test was performed three consecutive times interspersed with a passive recovery period of three minutes [23]. The best attempt was computed for analysis purposes according to the procedure by Mello et al., [23].

The hand grip test was also used to check the strength of the upper limbs, the same was done using a hydraulic dynamometer (JAMAR®, Cambuci, Brazil; calibrated before each assessment) [24]. The subjects remained seated on a bench with adjustable height and the forearm flexed at an angle of 90° [24]. All participants performed three maximum voluntary contractions (3 sec./duration) with their right and left hands, interspersed with recovery periods of 60 seconds, and the best performance was adopted for statistical analysis, according to the procedure Reijnierse et al., [24].

#### Analysis of neuromuscular performance of the lower limbs

The performance of the lower limbs was analyzed by tests of vertical jump (VJ) and jump against movement (CMJ), both jumps were analyzed through a force platform (CEFISE^®^, São Paulo, Brazil). Thus, the protocols established by Forza and Edmundson [25] were used. Before the evaluations, the volunteers performed a jump of each type to familiarize themselves with the tests, seeking to reduce errors during the execution of the protocols [25]. Then, starting from an orthostatic position, held for three seconds, with the knees flexed at approximately 90° and the hands fixed on the waist, the volunteers were instructed to perform the vertical jump as high as possible [25].

For CMJ analysis the same recommendations were adopted, however, the volunteers performed a squat followed by the jump [25]. A 10-minute recovery interval was established between VJ and CMJ [25]. For both tests, three attempts were made, interspersed with 60 seconds of passive recovery and the best attempt was adopted for data analysis, according to the procedure by Forza and Edmundson [25]. It is noteworthy that the height of the jumps (VJ and CMJ), were calculated by the force platform using the following formula [25]:

Jumpheight=t2*g*8‐1

t = flight time in seconds, g = gravity acceleration, with a value of 9.81 m / s^2^

#### Specific performance evaluation—experimental design

A distance of 100-m was established for the Sprint test, considering that this is the average distance achieved in the space of 20 seconds in tests performed on an indoor digital paddle ergometer [19]. After a general warm-up (10 minutes) consisting of a circuit of movements performed with body weight, the athletes performed a 100-m time trial on an indoor rowing type ergometer (Concept® model-D equipped with PM5 digital monitor, Florida, United States). The test was carried out in an air-conditioned environment (26°C). The equipment was calibrated with a resistance factor of 120 for females and 125_(N.s^2^/m^2^)_ for males according to the specifications of the international rowing federation. At the end of the tests, the results of the peak power in watts and test time in seconds were assimilated from the equipment by a computer attached to its digital monitor PM5.

After a 72-hour wash-out, the volunteers again underwent a general body warm-up (10 minutes) and immediately afterwards performed a 100-m time trial in an aquatic environment. The test was performed on a Single Scull vessel (model J9 brand Cucchietti^®^, Buenos Aires, Argentina; weight of 14 kg with a maximum weight support capacity of 85 kg; 820 cm in length and 29 cm in width) in open waters located at the headquarters of the Sport Club (The banks of the Potengi River) the ambient temperature was 26°C (We used a digital thermometer for the outdoor environment). Therefore, the water test was carried out at a specific time without significant climatic influences (i.e., winds; river current) previously verified at the Brazilian navy's hydrography center—tide tables captain of sea ports (Natal-RN; Brazil).

The Single Scull vessel was equipped with a NK-Sports® (Washington, United States) GPS (Speed coach model-2), connected to a portable computer via bluetooth for real-time monitoring through software (NK Link) which was programmed in the work option for a distance of 100 m. The equipment's motion sensor identified the boat's start which occurred automatically, when the 100-m was completed the equipment emitted an audible signal to inform the rowers that the test was finished, and the test time score in seconds was saved in the computer's memory. The volunteers were instructed to exclude any type of vigorous activity from the routine in the 72 hours preceding the specific tests mentioned above. It is important to note that no additional loads were added during the test in an aquatic environment.

All tests were performed at the same time at 8 am, and under similar climatic conditions in relation to the ambient temperature of 26°C.

#### Peak power analysis in terrestrial environment

The peak power in terrestrial environment was acquired by the indoor rowing type ergometer (Concept^®^, Florida, United States).

#### Peak power analysis in aquatic environment

To acquire the data of the peak power in an aquatic environment, the present study used a mathematical formula designed in a theoretical model by the authors. Initially, the variables were grouped by the similarity of the patterns by statistical analyzes based on the unsupervised machine learning technique of K-mean clusters. Thus, the correlations between the variables and the peak power generated in an indoor rowing ergometer were found.

Therefore, we continued with the modulation of the variables that indicated significant correlations with the peak power analyzed on the ergometer and through a multivariate regression analysis, we verified the predictive capabilities of the variables. Thus, the theoretical model was subsequently tested by means of a confirmatory factor analysis and by the reproducibility index in relation to the peak power obtained by the indoor ergometer digital ergometer. The variables used in the equation were: the displacement in meters per second, the distance covered in meters, the duration of the test in seconds, the total body weight and the total weight of the equipment (in this case, the vessel) in kilograms.

#### Statistics

The normality of the data was tested by the Shapiro-Wilk tests and Z-score of asymmetry and kurtosis (-1.96 to 1.96). The confounding factors sex (male and female) and chronological age present in the sample, were controlled during the regressions, Backdoor arithmetic was used to inhibit the effect of confounding factors during statistical analysis [26, 27]. The homogeneity of the regression models was tested by the Breush-Pegan test and the assumptions of normality, variance and independence of the data were not denied. To test the multicollinearity of the regression models, the Durbin Watson test was used. The data correlations were performed using the Pearson test. For the partial correlations, we controlled the effect of the lean mass variable, thus the magnitude used was that of Schober et al., [28]: Insignificant: r <0.10; Weak: r = 0.10–0.39; moderate: r = 0.40–0.69; Strong: r = 0.70–0.89; Very strong: r = 0.90–1.00. To measure reproducibility and reliability, the methods performed the calculation of the intraclass correlation coefficient and the magnitude used was determined by Miot [29]: absence: ICC = <0; poor: ICC = 0–0.19; weak: ICC = 0.20–0.39; moderate: ICC = 0.30–0.59; substantial: ICC = 0.60–0.79; and almost complete: ICC = ≥ 0.80.

The Bland and Altman [30] method was performed to verify the dispersion of the data within the limits of agreement defined by the differences in the means between the measures of the variables. Comparison analyzes ([terrestrial power peak x aquatic power peak]; [terrestrial sprint X aquatic sprint]) were performed using the Student-dependent t test. The effect size was calculated by the Cohen test (d), the adopted magnitude was [31]: insignificant: <0.19; small: 0.20–0.49; average: 0.50–0.79; large: 0.80–1.29; very large: <1.30. For the technical error of anthropometric measurements, the following magnitude was used: Acceptable for skinfolds ≤ 5.0%; Acceptable for other anthropometric measurements ≤ 1.0% [32]. All analyses were performed using open source software R (version 4.0.1, R Foundation for Statistical Computing®, Vienna, Austria) considering the significance of p <0.05.

## Results

Table 1 characterizes the sample of the present study. The individuals presented somatic maturation after peak growth speed, low amount of body fat and a strong concentration of lean mass. The margin of possibility of error pointed out for the sample size was a α equivalent to 4.90%, being below 5%, which suggests 95% reliability in the results obtained based on the sample used. It is worth mentioning that there were no sample losses. For all anthropometric variables, the technical error of measurement was below 1%.

**Table 1:** Sample characterization.

Variables	Values
n°(%)	16 (100%)
Male	13 (81%)
Female	03 (19%)
Age (years)	15.7 ± 1.21
Somatic maturation	1.79 ± 1.44
Height (Cm)	169.0 ± 9.59
Wingspan (Cm)	160.42 ± 44.8
Body weight (Kg)	64.7 ± 15.0
Body mass index (m^2^)	22.3 ± 3.63
Fat mass (kg)	16.1 ± 6.87
Lean mass (kg)	46.0 ± 9.63
Hand Grip (Kgf)	36.5 ± 8.04
Medicineball test (Cm)	4.75 ± 1.02
Vertical jump(Cm)	30.9 ± 7.43
Countermovement jump (Cm)	31.5 ± 8.50
Peak power (watts)**/** indoor rowing	331.1 ± 100.3
Peak power (watts)/Mathematical model	331.6 ± 104.1

The velocity in meters per second, the execution time in seconds, the total body weight and the mathematical model for the analysis of the peak power showed significant correlations with the peak power performed on the digital indoor rowing. Somatic maturation only showed a significant relationship with the peak of the force curve analyzed by the mathematical model proposed by the present study (see Table 2).

**Table 2:** Correlations of variables with the peak of the force curve.

Variables	Peak power in indoor rowing (Watts)		
	r	r^2^	p Value
Velocity (m / s)	0.926*	0.857	<0.0001
Run time (Sec)	-0.925*	0.855	<0.0001
Body weight (Kg)	0.817*	0.667	0.0001
Somatic Maturation	0.396	0.156	0.12
Peak power (Watts) /Mathematical model	0.891*	0.793	<0.0001
	**Peak power (Watts) /Mathematical model**		
Somatic Maturation	0.575*	0.330	0.01

* statistically significant.

The variables velocity, execution time and body weight exposed in regression model 1, are able to predict the performance of the peak power in a high precision (in 98%) (Table 3). Multicollinearity was not observed in relation to model 1.

**Table 3:** Regression model of variables with predictive capacity for the equation of the peak power in an aquatic environment.

Regression Model	r^2^ Adjusted	Β	p Value
1) Velocity (m / s)			
Runtime (Sec)	0.987*	8.06	<0,0001
Body weight (Kg)			

* statistically significant.

Table 4 shows the equation proposed by the present study for the analysis of the peak power (Watts) by the specific test performed in an aquatic environment. To estimate the peak power, the variables body weight (Kg), equipment weight (Kg) and speed (m / s) were used in which the test (in the case of 100-m) was performed, as shown in the Table 4.

**Table 4:** Predictive formula for the analysis of the peak power (watts) by the test performed in an aquatic environment.

**Mathematical model for peak power (watts)**
**Vel (m/s)** = DC (m) / t (s)
**Peak power (Watts)** = [(BW(Kg) + WE(Kg)) *Vel (m/s)]– 22

Vel (m / s) = Velocity in meters per second; DC (m) = Distance covered in meters; t (s) = Execution time in seconds; Pfc (Watts) = Peak power curve in watts; BW (Kg) = Body weight in kilograms; WE (Kg) = Weight of the equipment in kilograms.

**Fig 2:**
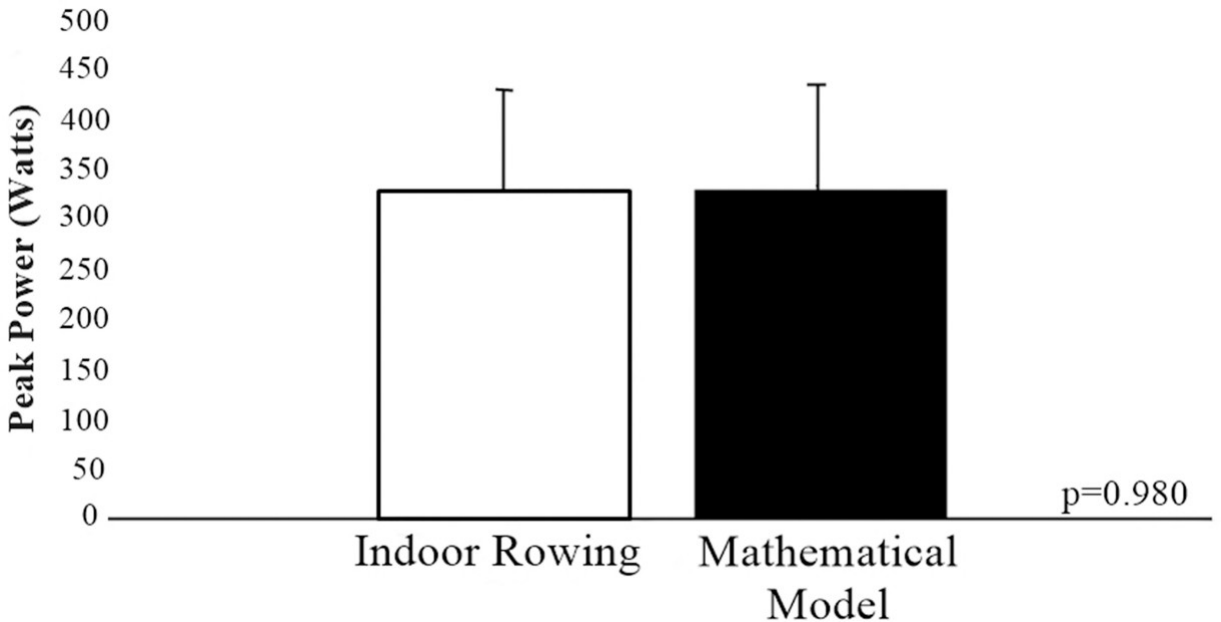
Comparison between the peak power of the indoor rowing with that acquired by the mathematical model proposed by the present study.

There was no statistical difference in the comparison between the peak power analyzed by the indoor rowing ergometer and the mathematical model proposed by the present study (Effect Size: -0.03; standard error indoor rowing = 25.08; standard error mathematical model = 26.03; p = 0.98). In addition, the reproducibility index of the intraclass correlation coefficient pointed to a high degree of reproducibility between the methods (ICC = 0.897; 95% CI = [0.737; 0.962]; F (15.16) = 18.4; p <0.0001) (Fig 2).

**Fig 3:**
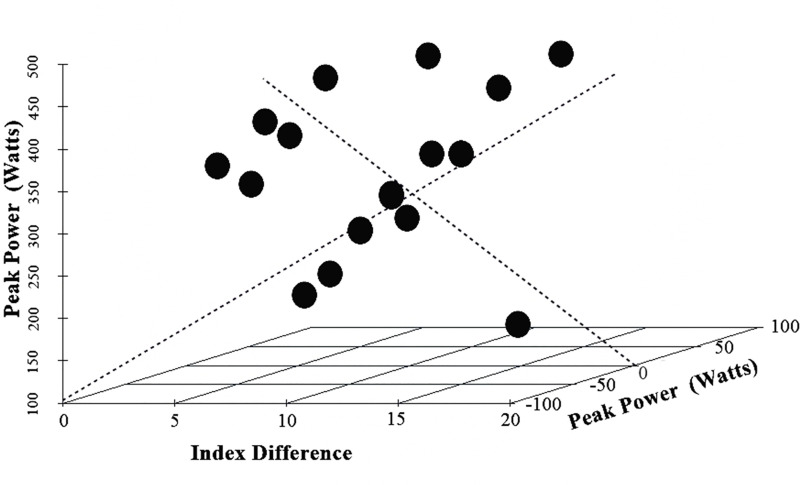
Bias proportion.

Through the Bland-Altman method, a significantly positive agreement limit (between -0.5 and 0.5; CI 95%: [-10.0; 10.0]) was found for the results of the methods used to assess the peak power (watts). Thus, the mathematical model developed by the present study did not point out any significant proportion bias (difference between the methods = 0.54 ± 47.8; r^2^ = 0.020; β = -0.330; CI 95% β: [-1.415; 0.754]; p = 0.550), which suggests a significant effectiveness of the mathematical model (Fig 3).

Considering that the 100m sprint was the distance used by the present study to determine the peak power, we sought to understand which variables would be associated with the 100-m performance. Thus, Table 5 shows significant and negative correlations between anthropometric variables, strength curve, neuromuscular performance of upper limbs and lean mass with sprint performance in both conditions. The negative relationships indicate that as the neuromuscular performance and peak power increase, there is a reduction in sprint time. That is, the greater the force and the peak power the faster the sprint.

**Table 5:** Correlations of variables with sprint performance.

Variables	Sprint indoor rowing			Sprint Single Scull		
	r	r^2^	p Value	r	r^2^	p Value
Somatic maturation	-0.300	0.090	0.2	-0.380	0.140	0.1
Lean mass (kg)	-0.900*	0.810	<0.0001	-0.790*	0.620	0.0002
Vertical jump (cm)	-0.490	0.240	0.07	-0.530*	0.280	0.04
CMJ (cm)	-0.370	0.130	0.1	-0.390	0.150	0.1
Medicineball test (cm)	-0.650*	0.420	0.006	-0.660*	0.430	0.004
Hand Grip (kgf)	-0.650*	0.420	0.005	-0.720*	0.510	0.001
Peak Power Indoor R (watts)	-0.990*	0.980	<0.0001	-0.880*	0.770	<0.0001
Peak Power Mm (watts)	-0.860*	0.739	<0.0001	-0.900*	0.810	<0.0001
Weight (kg)	-0.810*	0.650	0.0001	-0.810*	0.650	0.0001
Height (cm)	-0.760*	0.570	0.0006	-0.620*	0.380	0.009

CMJ = Countermovement jump test. Peak Power Indoor R = Peak Power in indoor rowing. Peak Power Mm = Peak Power in Mathematical model.

* statistically significant.

Similarly, it was observed in relation to the morphological variables (lean mass, weight and height). However, it is observed that although maturation was estimated by morphological variables, it did not show a significant relationship with the performance of sprints.

The lean mass showed a strong association with the sprint performance in rowers (Table 5). Thus, to verify the importance of this relationship, we controlled the effect of lean mass during associations with sprint performance (Table 6). In this sense, when controlling for lean mass, we showed that, with the exception of the peak power, all the relationships of the variables with the sprint disappeared in both conditions. Thus, the relationships were positive, indicating that as the performance of the variables increased, the sprint time also increased (Table 6).

**Table 6:** Correlations controlling the effect of lean mass on the sprint.

Variables	Sprint indoor rowing			Sprint Single Scull		
Lean Mass Variable Effect Control						
	r	r^2^	p Value	r	r^2^	p Value
Vertical jump (Cm)	0.150	0.020	0.5	0.130	0.010	0.8
CMJ(Cm)	0.170	0.020	0.7	0.150	0.020	0.1
Medicineball test (Cm)	0.220	0.040	0.4	0.30	0.090	0.2
Hand Grip (Kgf)	0.220	0.040	0.4	0.260	0.060	0.3
Peak Power Indoor R (watts)	0.990*	0.980	<0.0001	0.620*	0.380	0.01
Peak Power Mm (watts)	-0.040	0.000	0.8	-0.400	0.160	0.1
Weight (Kg)	0.090	0.000	0.7	0.360	0.120	0.1
Height (Cm)	0.400	0.160	0.1	0.140	0.010	0.6

CMJ = Countermovement jump test. Peak Power Indoor R = Peak Power in indoor rowing. Peak Power Mm = Peak Power in Mathematical model.

* statistically significant.

Table 7 shows the regression models that try to explain the sprint performance in the studied sample, and according to the data, both model 1 which includes the peak power measured by indoor rowing and model 2 which includes the peak power measured by mathematical model were able to predict sprint performance by more than 90%. It is noteworthy that there were no multicollinearities in relation to models 1, 2, and 3 (Table 7).

**Table 7:** Linear regression adjusted to estimate sprint performance in rowers.

Regression Model	r^2^ Adjusted	β	p Value
1) Lean mass (Kg)			
Medicineball test (Cm)			
Hand Grip (Kgf)	0.927*	-1.921	<0.0001
Height (Cm)			
Weight (Kg)			
Peak Power /Indoor Rowing (watts)			
2) Lean mass (Kg)			
Medicineball test (Cm)			
Hand Grip (Kgf)			
Height (Cm)	0.915*	-0.080	<0.0001
Weight (Kg)			
Peak Power /Mathematical model(watts)			

* statistically significant.

**Fig 4:**
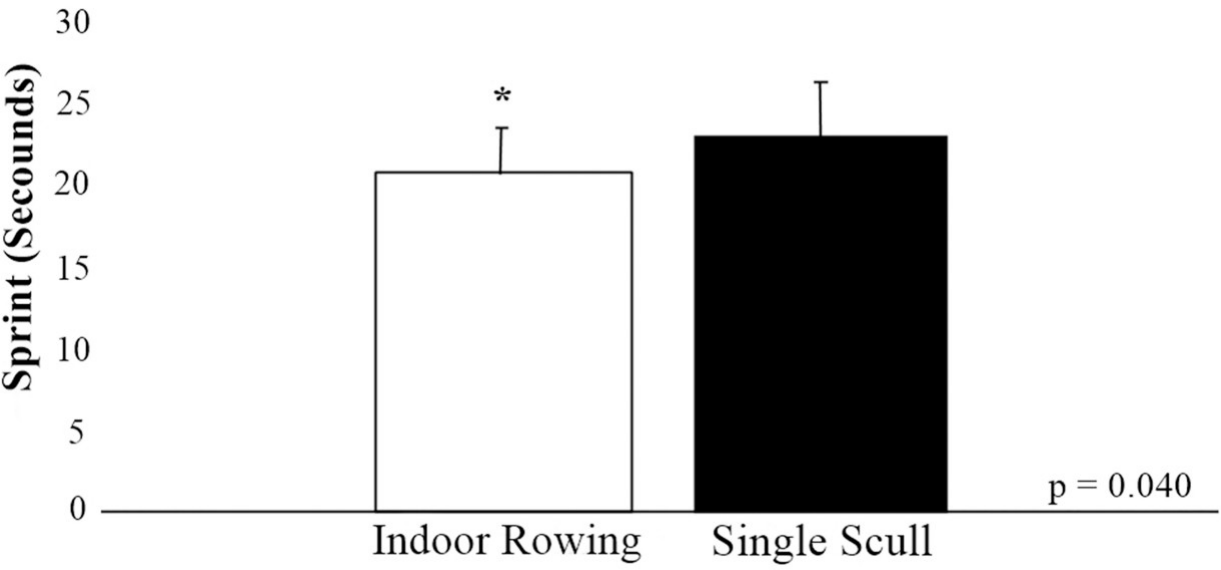
Comparison between sprint conditions. * statistically significant.

It is worth mentioning that for the reproducibility index between the tests performed on the Indoor Rowing and in the aquatic environment on the single scull vessel, a substantial intraclass correlation coefficient was indicated (ICC = 0.695; 95% CI [0.332; 0.881]; F (15.16) = 5.56; p = 0.0007). Nevertheless, in the comparison between the conditions of the sprints, the performance on the Indoor Rowing was statistically superior to the condition on a single scull vessel, thus the laboratory test lasted less time than the specific test in water (Effect Size: 0.73; standard error sprint Indoor Rowing = 0.65; standard error aquatic sprint = 0.83; p = 0.04) (Fig 4).

## Discussion

The objective of the study was to develop an equation to provide the peak power in rowers through a specific stimulus performed in an aquatic environment, as well as to correlate morphological, anthropometric and strength variables with rowing performance. The main findings of the present study were: (i) A significant reliability, between the peak power provided by the mathematical model and that provided by Indoor Rowing. (ii) Maturation was related to the peak power in an aquatic environment. (iii) The morphology, anthropometry and strength of the upper limbs were related to the sprint and peak of the power in both tests. (iv) The sprint tests in the aquatic environment and indoor rowing showed significant reliability between them.

This is the first study to propose an equation that provides a direct measure of a rowers' peak power in the aquatic environment. According to the literature, previously Riechman et al., [18] and Cataldo et al., [19] developed mathematical models aimed at predicting the performance of 2.000-m from tests of 30-s and 20-s respectively. Both authors presented tests performed on an ergometer, and the tools do not show reproducibility in an aquatic environment, and in relation to the peak power, no mathematical model or specific test for this variable was presented by any of the referred authors.

Taking into account that new techniques with practical applicability in monitoring the performance of rowers are encouraged [4]. Although the tests were carried out in a controlled environment, the mathematical model presented by the present study can be applied under conditions similar to the specific ones imposed by the aquatic environment (i.e., winds, water resistance, sea current). Moreover, this can mitigate against the need for laboratory resources and equipment that have a relatively high financial cost for athletes and or competitive clubs.

In addition, the tool outlined in this research can be used to classify the athletes' current stroke power profile (i.e., stroke or bow), taking into account the peak power in the aquatic environment. In this way, it becomes simple to direct the training of these subjects according to the characteristics of their rowing power profile, or to group rowers in a collective boat for team competitions from their individual power profile, or even to calculate the peak power of team boats.

In addition, among the factors associated with the production of maximum power and muscle strength in aquatic and land sports are lean mass and total body weight [33–35]. This fact corroborates the findings of the present study, where although the sprint time in the aquatic environment was shorter in relation to the terrestrial environment, there was a significant association between body weight and lean mass in relation to the performance in the 100 m sprint at the peak of rowers' power in water and indoor rowing (p <0.05). It is important to note that in the present study the difference observed between sprints (terrestrial and aquatic), can be explained by the hydrodynamic influence of the boat's hull on the liquid [10, 12–14].

In addition to the morphological characteristics Hatchett et al., [36], highlight that the performance of rowing over short and long distances (100-m and 2,000-m) seems to be influenced by physical attributes such as the specific muscular strength of the upper limbs. This information is in accordance with the data from the present study, which found a significant association between upper limb strength performance and indoor rowing and aquatic sprint (p <0.005; Table 4). In relation to muscle strength, previous studies have identified that biological maturation influences the strength of young rowers and young athletes [3, 8, 37, 38]. This fact can be observed in the present study, where a significant association was found between the peak power in the aquatic environment and somatic maturation (r^2^ = 0.330; p = 0.01).

In the aquatic environment, generating a peak of power that makes it possible to increase the speed of the vessel's locomotion, can give advantages in sports events [1, 3, 10]. However, it will require a large production of traction force generated from the movements performed by the rowers, and having higher concentrations of lean mass can favor the production of force [1, 3, 10, 39]. Concomitantly the maturation process has a significant relationship with the lean mass and thus also with the performance of young rowers [38]. The results of the present research showed that when statistically controlling the effect of lean mass on the relationship between upper and lower limb muscle strength performance with indoor rowing and aquatic sprint performance, the significant correlations disappeared (Table 6). Thus, it was evident that lean mass has a significant influence on the performance of rowers.

Recently, our group investigated the interaction of maturation and lean mass with the neuromuscular performance of young elite athletes [39]. It was shown that lean mass significantly interacts with the neuromuscular performance of upper and lower limbs of young Brazilian elite rowing athletes [39]. This fact corroborates the findings of the present study in relation to lean mass. In addition, although maturation has been associated with higher concentrations of muscle strength [37, 40–42], we found that maturation was not associated with the peak power estimated by the mathematical model proposed by our study. Suggesting that there is no need to take maturation into account when estimating peak power.

In view of all the aforementioned aspects, it is noteworthy that in addition to directing the training, the specificity of the task is useful for bringing the rowing athlete closer to the real challenge that will be found in a sports competition [5]. Therefore, the mathematical model developed by the present study will be extremely important for countries with a tropical climate, where it is possible to perform specific tests at any time of the year, instead of countries where the winter is more extreme and would prevent the execution of tests in specific environment in a certain period of the chronological year, thus impairing the frequent monitoring of athletes' sports performance.

Despite the relevance of this study, some limitations were observed: (i) Biological maturation was evaluated using a predictive model, the results based on the gold standard (hand and wrist X-rays and longitudinal monitoring during puberty) may be different in relation to the maturation stage; (ii) the study design was observational, which did not allow us to establish a cause and effect relationship in the correlation analyzes.

In view of the results presented by the present study, the equation of the peak of the aquatic power brings opportunities for future uses including the possibility of monitoring the strength performance during a competition, allowing analysis of the peak power achieved between different races. This would enable coaches to instigate a change in strategy if necessary. In addition, the mathematical model presented in this research can be used to estimate the peak power in advance. In this context, coaches can prescribe time targets for his athletes in order to achieve the peak of power necessary for the displacement within the shortest desired time.

## Conclusion

It is possible to conclude that the equation for the peak power in an aquatic environment is highly reliable with analyses performed on the indoor rowing digital ergometer. In addition, somatic maturation, neuromuscular and anthropometric variables are related to the sprint performance of 100 meters and the peak of the Junior’s rowers' power.

## Supporting information

S1 Raw data (XLSX)
